# Characterization of early cortical population response to thalamocortical input *in vitro*

**DOI:** 10.3389/fnins.2013.00273

**Published:** 2014-01-31

**Authors:** Michael R. H. Hill, Susan A. Greenfield

**Affiliations:** Department of Pharmacology, University of OxfordOxford, UK

**Keywords:** thalamocortical, *in vitro*, mouse, barrel cortex, voltage sensitive dye, local field potential

## Abstract

The *in vitro* thalamocortical slice preparation of mouse barrel cortex allows for stimulation of the cortex through its natural afferent thalamocortical pathway. This preparation was used here to investigate the first stage of cortical processing in the large postsynaptic dendritic networks as revealed by voltage sensitive dye imaging (VSDI). We identified the precise location and dimensions of two clearly distinguishable dendritic networks, one in the granular layer (GL) IV and one in the infragranular layer (IGL) V and VI and showed that they have different physiological properties. DiI fluorescent staining further revealed that thalamocortical axons project on to these two networks in the typical barrel like form, not only in the granular but also in the IGL. Finally we investigated the short-term dynamics of both the VSDI signal and the local field potential (LFP) in response to a train of eight-pulses at various frequencies in both these layers. We found evidence of differences in the plasticity between the first two response peaks compared to the remaining six peaks as well as differences in short-term plasticity between the VSDI response and the LFP. Our findings suggest, that at least early cortical processing takes place in two separate dendritic networks that may stand at the beginning of further parallel computation. The detailed characterization of the parameters of these networks may provide tools for further research into the complex dynamics of large dendritic networks and their role in cortical computation.

## Introduction

*In vitro*, stimulation of the thalamocortical afferents, even at high stimulus amplitudes, leads to voltage sensitive dye imaging (VSDI) activity confined to two layers only in the rodent primary somatosensory whisker cortex (S1, Higashi et al., 1999; Laaris et al., 2000; Itami et al., 2001; Laaris and Keller, 2002; Llinás et al., 2002; Urbano et al., 2007). This spatial confinement is in part due to the fact that in brain slice recordings *in vitro*, polysynaptic propagation and general background neuronal activity is attenuated because of the large number of severed neuronal projections and the absence of neuromodulators (Hájos and Mody, 2009). For example, voltage-sensitive glutamatergic NMDA receptors of layer III function as coincidence detectors. In the absence of the continuous barrage of subthreshold excitatory postsynaptic potentials *in vitro*, they therefore fail to respond to single pulse stimulation of the thalamus (Laaris et al., 2000). Beyond these general limitations of the *in vitro* setup, the strong local inhibitory physiology of S1 will probably also actively contribute to the spatial limitation of the response.

In rodents primary sensory whisker information is relayed from the whiskers through brainstem nuclei onto neurons in the ventral posterior medial part of the ventral basal nucleus of the thalamus (VPM). This sub-nucleus consists only of excitatory thalamocortical neurons, which project into cortical layer IV and form collaterals into layer Vb and layer VI as they ascend through cortex (Pierret et al., 2000). In layer IV of S1 approximately 10–25% of all synapses originate from these thalamocortical afferents (Benshalom and White, 1986). The target cortical neurons consist of approximately 75% excitatory neurons (Ren et al., 1992), the remaining 25% are inhibitory neurons which display radial axonal projections with a confined radius of less than approximately 200 μm with recurrent loops back into a locally confined physiological structure called “barrel” (Porter et al., 2001,these barrel-like structures give S1 its alternative name: barrel cortex, Woolsey and Van der Loos, 1970). Groups of these inhibitory neurons can also inhibit each other and are interconnected by gap junctions, resulting in highly synchronized, amplified and locally confined feedforward and feedback inhibitory networks (Gibson et al., 1999; Porter et al., 2001; Beierlein et al., 2003; Tan et al., 2008).

The general *in vitro* limitations and this strong local inhibitory network are the reasons for the spatially restricted VSDI response in the *in vitro* barrel cortex. Here we investigate in detail the characteristics and short-term plasticity of this response. The thalamocortical synapse is a key component in many functional theories of brain processing. Besides its obvious role in sensation, these range from the mechanisms of anesthetics and dreaming (Franks, 2008) as far as to the functional basis of cognition (Llinás et al., 2002). Determining the nature and characteristics of the postsynaptic population response in the barrel cortex can provide valuable insight into the mechanisms underlying the orchestrated activity of extended dendritic networks acting together to process primary sensory input to cortex.

## Materials and methods

### Electrophysiology

In accordance with the United Kingdom Animal (Scientific Procedures) Act of 1986, this study did not require a Home Office project license because no regulated procedures were carried out. Male CB57BL/6 mice, p18–p21 (Harlan Laboratories™, Bicester, UK), were humanely killed at a designated establishment by overdose of anesthetic, which is an appropriate method under Schedule 1 of the Act. The brain was carefully removed and cooled down in artificial cerebrospinal fluid (aCSF) at 0°C for approximately 1–2 min. aCSF was prepared and stored as two separate stock solutions with the final solution containing: 124 mM NaCl, 5 mM KCl, 1.25 mM NaH_2_PO_4_, 1.25 mM MgSO_4_, 2 mM CaCl_2_, 10 mM glucose, and 22 mM NaHCO_3_ at pH 7.4 (±1). The brain was then blocked according to Agmon and Connors (1991) and 400 μm thick slices were sectioned with a vibratome (VT 1000S, Leica™ Microsystems, Milton Keynes, UK) and transferred to oxygenated aCSF at room temperature.

Di-4-ANEPPS (Invitrogen™, Molecular Probes™, Paisley, UK) voltage sensitive dye stock solution was made up at a 6.93 mM in 7% (v/v) cremophore EL (Sigma–Aldich™ Ltd., Dorset, UK) in DMSO. The final dye solution was made up on the day of experimentation by adding 50% (v/v) oxygenated aCSF to fetal bovine serum with a total resulting volume of 1.5 ml. To this 80 μl di-4-ANEPPS stock solution were added resulting in a final concentration of 0.35 mM. This dye solution was kept in a 5 ml Eppendorf tube topped up with 95% O_2_/5% CO_2_ and vortexed for 3 min to ensure adequate oxygenation before application to slices.

After incubation in oxygenated aCSF, the brain slices were placed on filter paper submerged in oxygenated di-4-ANEPPS solution. They were protected from light at room temperature for 30 min. After dye internalization slices were rinsed with aCSF and returned to the incubation chamber. The incubation chamber was then placed into a light protected water bath where the slices were slowly warmed up to 32°C until transferal into the actual recording chamber.

After incubation for 30–60 min, an individual slice was moved to a membrane chamber for recording (Hill and Greenfield, 2011). A concentric bipolar tungsten micro electrode (CBARC75, FHC™, Bowdoin, USA) was then placed into the VPM. Two monopolar tungsten microelectrodes (UEWMEFSEBN3M 10/60/0, FHC™, Bowdoin, USA) were used for recording the local field potential (LFP) within layer IV and the boundary of layer Vb and layer VI. The slices were left to equilibrate in the recording chamber with the electrodes in place for 20 min prior to experimenting. The recorded LFP signals were amplified 10× in a Neuro Data IR-283 amplifier (Cygnus Technology™, Delaware Water Gap, USA) and amplified again 100× in an in-house custom made amplifier and low pass filter module (1 kHz cutoff, Preston, M, Department of Pharmacology, Oxford, UK).

LFP data was recorded with the Signal™ software in combination with the Micro 1401 mkII AD/DA converter (both from Cambridge Electronic Design™, Cambridge, UK). VSDI images were acquired with a Brainvision™ MICAM02 system and Analyzer software (Brainvision™ Ltd, Tokyo, JP, SciMedia™ Ltd, Costa Mesa, USA) as well as an in-house custom made inverted microscope (Hill, M.R.H., Department of Pharmacology, Oxford, UK). All recorded data was analyzed in Matlab™.

The VSDI signal was averaged over 25 recordings and savitzky-golay filtered (size: 7, order: 1) with each recording lasting 850 ms at a frame rate of 1 ms/frame. The LFP was baseline subtracted as to remove any line noise and also averaged over 25 recordings. Facilitation was measured in the LFP as the slope across the mean of the amplitude of the individual peaks minus the amplitude of the individual signal just before the respective artifact. In the VSDI signal facilitation was measured in the same way but relative to the local minimum between the current and the previous peak.

Unless otherwise indicated all statistical analysis was based on a *t*-test or a repeated measures ANOVA respectively. Whenever sphericity was violated a Greenhouse–Geisser correction was applied (GGε). Bonferroni or Holm–Sidak *post-hoc* correction was applied to correct for multiple comparison at an alpha-level of 0.05. Trial numbers (*n*) refer to the number of slices from which data was recorded. Per animal no more then two slices were included in any analysis, one from each hemisphere.

### Histology

Slices for staining were re-sliced to a thickness of 100 μm. Cytochrome oxidase (CO) staining required that solution was made up fresh on the day of staining. 0.5 mg/ml DAB was dissolved in phosphate buffer (PB) at 50–55°C and 0.3 mg/ml cytochrome C was added followed by 0.04 mg/ml sucrose and stirred for 1 h. The slices were then immersed in the CO solution and left to incubate in the dark for 8–10 h at 37°C. When a good stain was achieved the slices were dehydrated and mounted (adapted from Wong-Riley, 1979, Land and Simons, 1985 by Grandy T., Department of Pharmacology, Oxford, UK).

For cresyl violet (CV) staining the 100 μm thick slices were first dehydrated and mounted. The slices were then placed in water for 1 min and the CV solution for 30 min or until they showed an even, homogeneous blue stain. Following staining, the slices were passed through distilled water, 50, 70, and 80% EtOH for 1–2 min each followed by 90% EtOH for approximately 15 min, depending on the degree of staining (adapted from Geisler et al., 2002 by Grandy T., Department of Pharmacology, Oxford, UK).

DiI crystals (Invitrogen™, Molecular Probes™, Paisley, UK) were gently lodged into the VPM of living 400 μm thick thalamocortical slices after VSDI. The slices were then fixed in 4% paraformaldehyde and left in the dark at room temperature for at least 4 weeks. Images were acquired at 573 nm (excited at 556 nm). Seventy-five to eighty-five images were recorded from each brain slice at increasing exposures and reassembled into a high dynamic range image in Photomatix Pro (HDRsoft™, Montpellier, France).

## Results

### Thalamocortical projections

**Figure 1:**
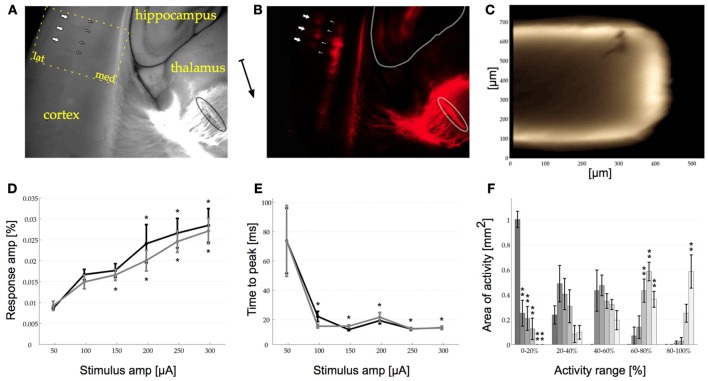
**(A)** Photo of part of the thalamocortical brain slice including cortex, hippocampus, and thalamus. The dotted outline marks the recording area for all VSDI data presented here (compare to Figures 2A,B, lat: lateral, med: medial). **(B)** Same as in **(A)** but imaged only at 573 nm (excited at 556 nm). A DiI crystal in the VPM is outlined in the bottom right corner [also in **(A)**] from where axon bundles ascend along subcortical white matter (thin vertical line in center of image) into cortex. In cortex two bands of DiI staining can be seen, the granular (GL, to the left) and the infra-granular layer (IGL, to the right). In both layers barrel structures can be recognized clearly as blobs. In the GL these are pointed out with thick arrows, whilst the barrel walls are pointed out with thin arrows [same in **(A)** where the barrel walls can also be seen in the normal light photograph]. The arrow between **(A)** and **(B)** indicates the approximate orientation of the midline pointing toward ventral.**(C)** Cross section of 600 μm thick slice stained with di-4-ANEPPS. The dye penetrates diffusely up to 75 μm into the slice (bright outline). **(D)** Response amplitude curve for VSDI response to single stimulation pulses in the GL (dark trace, asterisks on top) and the IGL (light trace, asterisks below). The response amplitude increases monotonically with increasing stimulation amplitude. The asterisks indicate significant (*p* < 0.05) difference from first response in Holm–Sidak *post-hoc* testing. GL (dark trace): *p*-values compared to 50 μA for 100–300 μA: *p*(0.205), *p*(0.044), *p*(0.006), *p*(0), *p*(0). IGL (light trace): *p*-values compared to 50 μA for 100–300 μA: *p*(0.013), *p*(0.008), *p*(0.001), *p*(0), *p*(0). **(E)** Time To Peak for the GL and the IGL [same format as in **(D)**] showing that the response is locked between 13 and 23 ms as soon as the stimulation amplitude reaches 100 μA. The response amplitude is measure relative to baseline. GL (dark trace): *p*-values compared to 50 μA for 100–300 μA: *p*(0), *p*(0), *p*(0), *p*(0), *p*(0). IGL (light trace): An ANOVA revealed that there were no significant differences between the 6 tested conditions, GG-epsilon corrected *p*(0.059) > 0.05. *p*-values compared to 50 μA for 100–300 μA: *p*(0), *p*(0), *p*(0), *p*(0), *p*(0). **(F)** Amount of area with VSDI signal within a certain range of the maximum response amplitude over all trials [0–20, 20–40, 40–60, 60–80, and 80–100% range, different shades of gray correspond to trials in **(D)** and **(E)**]. The amount of area with less than 20% activity decreases monotonically (left side) whilst the area with high activity increases monotonically with stimulation amplitude [same amplitudes as in **(D)** and **(E)** represented in dark to light gray bars], Bonferroni corrected Holm–Sidak *p* < 0.01. For visibility only the data from the GL is shown, the results in the IGL were similar. In **(D)**, **(E)**, and **(F)** error bars are s.e.m., *n* = 6. 0–20% all *p*-values *p*(0), *p*(0), *p*(0), *p*(0), *p*(0), 20–40%: *p*(0.104), *p*(0.273), *p*(0.645), *p*(0.314), *p*(0.36), 40–60%: An ANOVA revealed that there were no significant differences between the 6 tested conditions, GG-epsilon corrected *p*(0.066) > 0.01. *p*(0.867), *p*(0.439), *p*(0.592), *p*(0.279), *p*(0.785), 60–80%: *p*(0.5), *p*(0.183), *p*(0), *p*(0), *p*(0.001), 80–100%: p(1), *p*(0.877), *p*(0.757), *p*(0.009), *p*(0).

In all experiments we stimulated the dorsal part of the VPM to elicit activity in barrel cortex in mouse somatosensory thalamocortical brain slices. We used fluorescent DiI tracer to verify that our slicing protocol conserved thalamocortical connections between the VPM and S1 and to get a clearer understanding of the nature of these connections. DiI stained slices remained dark outside of the ventrobasal thalamus and the S1 region with the exception of low-level background fluorescence in the hippocampus in some slices. DiI staining was strongest in the ventrobasal area of the thalamus, where the crystals were initially inserted. From here bundles of axons could be seen projecting to subcortical white matter, which was stained prominently. Above white matter, in S1 gray matter, two bands of fluorescence could be seen. A brighter and longer band at the border of layer V/VI (the infragranular layer, IGL) and a weaker stained, shorter band in layer IV (the granular layer, GL), resembling the two layers of thalamocortical synapses that reach from the VPM into S1. Both bands were in-homogenous in their internal structure reflecting the local barrel-like anatomy of S1. These barrels were more clearly delineated in the GL, but could also be easily discriminated in the IGL (*n* = 3, Figures 1A,B).

### Dye penetration

After investigating the structure of the relevant and intact anatomy within our slices with DiI we wanted to know how deep VSD penetrates these slices. To do so, we re-sliced stained 600 μm thick thalamocortical brain slices orthogonally to their original slicing plain (*n* = 6). These cross sections revealed that the dye homogeneously diffuse into the tissue to a depth of no more than 100 μm. Beyond a depth of 50 μm, however, the concentration of the dye decreased rapidly. In view of this gradient in dye uptake and the fact that the VSDI signal has a low signal to noise ratio, we concluded that all measured responses were superficial, originating from a depth of up to approximately 75 μm (Figure 1C).

### Basic physiology in S1

The majority of published work so far has focused on VSDI responses in S1 to low levels of stimulation with the aim of activating only a single barrel. Here we investigated a population response across multiple barrels, which we believe may more closely resemble the barrage of information reaching S1 during sensory investigation of the real world. We focused on the response at the thalamocortical synapses in the GL and the IGL, as localized with DiI (see above). First, we recorded a VSDI response-amplitude curve ranging from 50 to 300 μA in both layers (Figure 1D). A One-Way repeated measures ANOVA revealed that an increase in stimulus amplitude monotonically increased the amplitude of the signal measured in the barrel cortex in response to single pulse stimulation of the VPM across all stimulation amplitudes (*n* = 6). This effect was seen both in the GL [*p*_(0)_ < 0.05] and the IGL [*p*_(0)_ < 0.05]. With increasing stimulation amplitude the recorded mean response amplitudes in the GL were: 0.009, 0.017, 0.018, 0.024, 0.027, and 0.029%. In the IGL these values were: 0.009, 0.015, 0.017, 0.02, 0.025, and 0.027%. Holm–Sidak group statistics showed a significant difference in both layers for all recordings above and including 200 μA (in the IGL already responses to stimulation at 150 μA were significantly different). At 50 μA the time at which the response reached its maximum amplitude after stimulation onset (time to peak) showed an inhomogeneous and wide distribution with a 95% confidence interval of 114.33 ms in the GL and 124.47 in the IGL, indicating that, if there was any response at all present in the cortex, its amplitude was below noise levels (Figure 1E). At 100 μA and above, the time to peak was locked in the range of 13–23 ms. This effect was found to be significant in the GL [GGε corrected *p*_(0.044)_ < 0.05] but in the IGL no significant difference could be shown [GGε corrected *p*_(0.059)_ > 0.05], which was probably due to the non-spherical nature of the data [Mauchly's test for sphericity in the IGL was highly significant: *p*_(0)_ < 0.01]. With increasing stimulation amplitude the recorded mean latencies in the GL were: 73.83, 22.17, 13.17, 19.33, 13.67, and 14.5 ms. The mean latencies in the IGL were: 73.67, 15.5, 15.5, 21.67, 13.83, and 14.33 ms.

Besides the time to peak and the mean amplitude across our regions of interest (ROIs, i.e., GL and IGL), we were also interested in the spatial distribution of activity within the two layers. Specifically we wanted to quantify whether at higher stimulation amplitudes the same area of activity simply increased its response amplitude or whether a larger area of cortex was activated and how this changed across different stimulation amplitudes. A quantitative analysis of the distribution of activity across all tested stimulation amplitudes revealed, that at 50 μA no pixels recorded response amplitudes higher than 40% of the maximum value across all conditions. The difference of the amount of area activated across different stimulation amplitudes reached a significant level in the 0–20% activity range [Bonferroni corrected *p*_(0)_ < 0.01] in the GL and [GGε and Bonferroni corrected *p*_(0) < 0.01_] in the IGL. In other words, at 50 μA activity across the whole ROI remained at, or close to, baseline values. As soon as the simulation amplitude was increased, however, this changed significantly resulting in a monotonically decreasing fraction of the ROI remaining below 20% activation (left side of Figure 1F), i.e., a large area of activation. A similar inverse effect could be seen in the activity range of 60–80% [Bonferroni corrected *p*_(0) < 0.01_] and the 80–100% range [GGε and Bonferroni corrected *p*_(0.004)_ < 0.01] although here the effect was only found to be significant in the IGL (right side of Figure 1F, *n* = 6).

This data shows, that the elicited response was detectable in the barrel cortex at amplitudes as low as 100 μA. The response amplitude, however, was only significantly different from noise (i.e., values at 50 μA stimulation amplitude as indicated by the 95% confidence interval range of the response) at 200 μA and above. The change in response amplitude was due to both a monotonic increase in the amount of area that was activated at higher stimulation amplitudes as well as a monotonic increase in response amplitude within the active area. On the basis of these findings we decided to conduct the following experiments with a stimulation amplitude of 200 μA.

### Dimensions of the GL and the IGL

**Figure 2:**
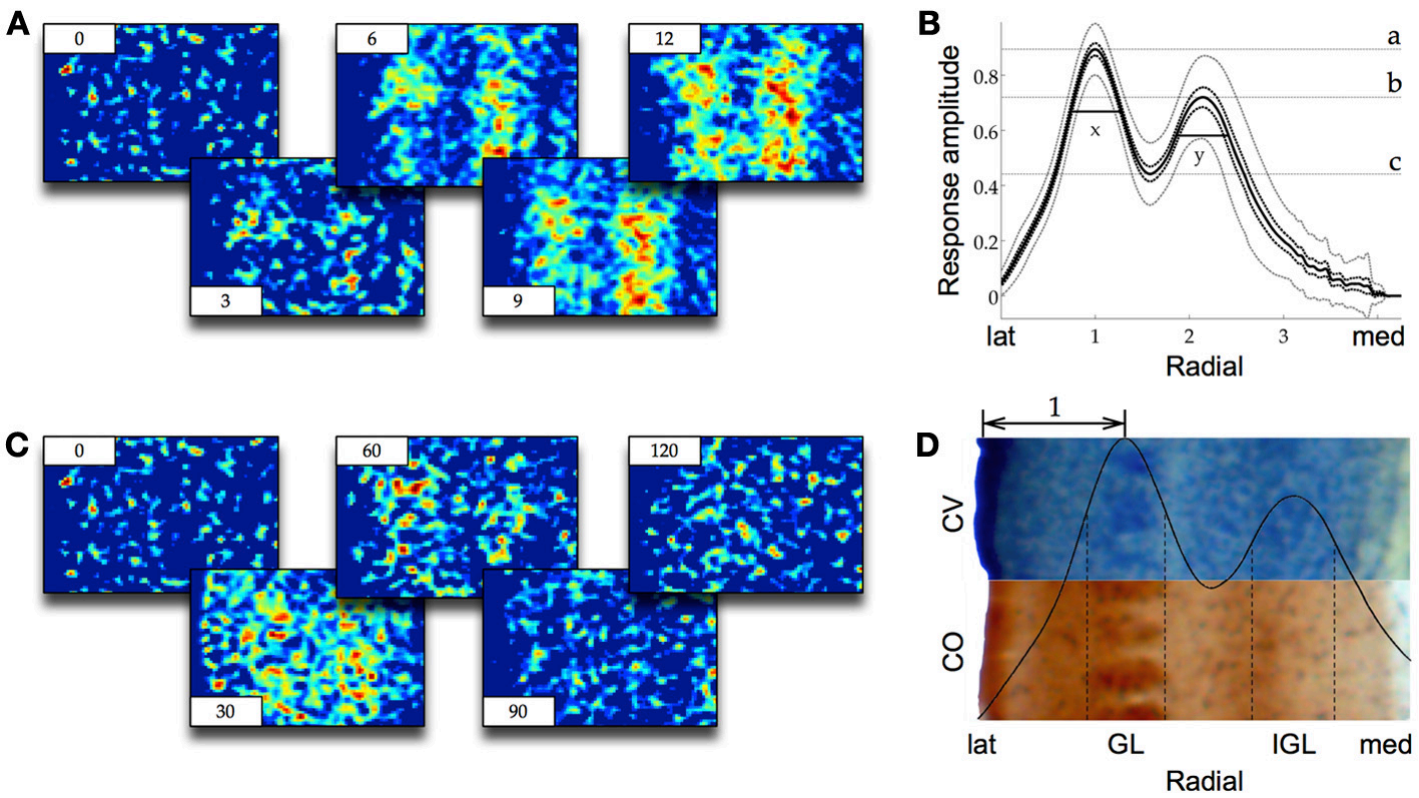
**(A)** Representative screenshots of VSDI recording in barrel cortex during stimulation of VPM (averaged over 25 trials, 1000 Hz frame rate, time in ms in corners, lateral to the left, see Figure 1A for orientation). Warmer colors from blue through green and yellow to red indicate higher response amplitudes. Two bands of activity (the GL on the left and the IGL on the right) appear and quickly reach their maximum amplitude over the course of the first few milliseconds (compare also to Figure 3). **(B)** Same as **(A)** but showing the decay of the signal over the course of 120 ms. **(C)** The response in **(A)** normalized and averaged along the y-axis and across frames 7–16, *n* = 20. Black dotted lines are the s.e.m., gray dotted lines are the 95% ci. Irregularities toward medial are artifacts due to discontinuous data at the proximal border of cortex. The two bands of activity can easily be distinguished as two local maxima (a and b), the GL on the left, the IGL on the right. The width of the two layers (x and y) are measured as the half maximal amplitude from the local minimum between them (c, see Results for details). **(D)** The same response profile as in **(C)** but now relative to a cresyl violet (CV) and a cytocrome oxidase (CO) stained histological preparation. The half maximal width of the GL and the IGL are delineated with dotted lines. The staining reveals that the barrel structures in layer IV are colocalized with the GL and the IGL lies at the border of LV and LVI. The double headed arrow is the standardized metric [same as x-axis in **(C)**].

*In vitro* stimulation of the VPM results in two bands of VSDI activity in S1 parallel to the cortical layers. In our recordings we observed these two bands of activity across multiple barrels. The signal in both layers increased rapidly over a course of 10 to 20 ms (Figure 2A) and then died away slowly over the following 100 ms (Figure 2B). In the literature the location of these two bands has been reported to be either within layer IV and layer VI eventually spreading to layer II/III and V (Higashi et al., 1999) or within layer IV and layer V/VI, without any further spread (Laaris et al., 2000; Itami et al., 2001 albeit in neonatal mice) or within layer IV with a spread to layer II/III and V (Llinás et al., 2002). To measure the precise location and dimensions of the two bands of VSDI activity we first computed a standard response profile. For this the response amplitude of all trials was normalized to 1 and the radial axis was normalized according to:

$$x_{\text{norm}}=\left(x-a\right)\left(\frac{1}{b-a}\right)$$

where *a* = Edge of the cortex *b* = First local maximum of the normalized response amplitude.

This normalization introduced a standard metric to the measured responses where the distance from the edge of cortex to the local maximum of the first band of activity was equal to *b*_norm_ − *a*_norm_ = 1. This metric allowed for VSDI data averaged across many animals to be put into relation to the varying anatomical dimensions of S1. Within this metric the location of the IGL was 2.14 times the standard metric from the edge of the cortex. The diameter of the GL (width at half maximal amplitude) was 0.54 times the standard metric and the diameter of the IGL was 0.56 times the metric (Figure 2C). Comparison to CV and CO stained slices showed that the GL was at the same location and of the same diameter as layer IV (Figure 2D). The IGL was located at the boundary between layer V and layer VI, reaching approximately half way into either layer, possibly with a slight bias toward layer VI. In the absence of any pharmacological manipulation we did not observe any spreading into adjacent layers in response to single pulse stimulation of the VPM. In response to pulse train stimulation we did observe a spread into more superficial areas as in Laaris et al. (2000, data not shown).

**Figure 3:**
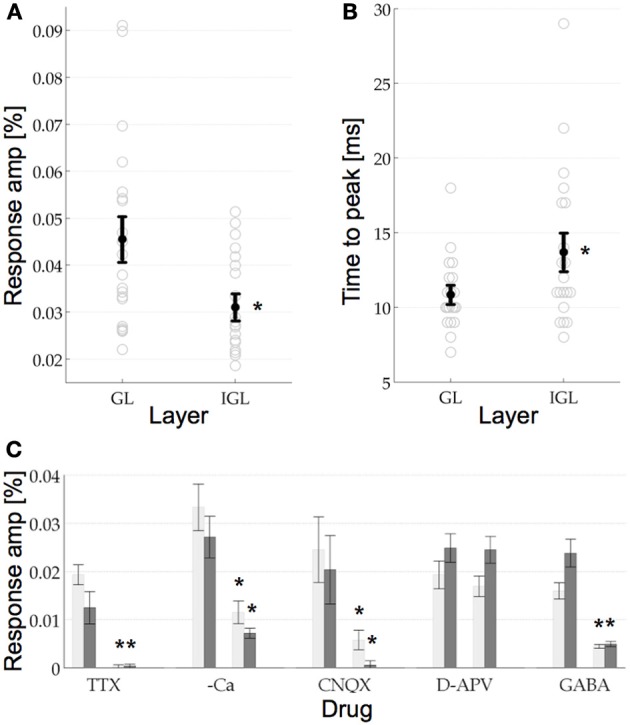
**(A)** VSDI response amplitude in the GL and the IGL. The response amplitude in the GL was 0.0456%, which was significantly higher than in the IGL at 0.0311%. Error bars are the s.e.m, circles are the raw data, *n* = 20. **(B)** Response time to peak for the two layers. Same format as **(A)**. The response in the GL reached its maximal response amplitude after 13.85 ms, which was significantly earlier than the response in the IGL at 16.7 ms. **(C)** Pharmacological manipulations in the two layers. *T*-tests revealed that the signal in the GL (light gray bars) and the IGL (dark gray bars) was reduced significantly by TTX, Calcium free aCSF (–Ca), CNQX, and GABA. Neither layer showed any significant response to D-APV. See Results for details, error bars are the s.e.m.

### Comparing GL and IGL physiology

After quantifying the anatomical dimensions of the GL and the IGL, we wanted to see if they also have different physiological properties. For this we compared the response amplitude and time to peak of the GL and the IGL. The response amplitude (mean and s.e.m.) in the GL was 0.0456% ± 0.0045, which was significantly higher than that in the IGL, 0.0311% ± 0.0025, *p*_(0.008)_ < 0.05 (*n* = 20, Figure 3A). Their response latencies were also significantly different. The signal first reached its maximum response amplitude in the GL after 13.85 ms ± 0.54 followed by the IGL after 16.7 ms ± 1.17, *p*_(0.034)_ < 0.05, (*n* = 20, Figure 3B). No significant difference between the two layers was found in respect to the time that the signal prevailed. In the GL the signal returned to baseline after 105.6 ms ± 6.6, in the IGL after 89.34 ms ± 9.36 (the return to baseline was measured as the point at which the response ±1.5 std returned to the mean response value measured in the pre stimulus interval ±1.5 std, data not shown, *cf*. discussion.).

### Pharmacology of the GL and IGL

Pharmacologically both layers showed the same properties (Figure 3C). The addition of TTX to the aCSF (bath applied) resulted in a complete extinction of the response, confirming that the signal was sodium channel dependent [in the GL *p*_(0.029) < 0.05_, and in the IGL *p*_(0.029)_ < 0.05]. The signal was also found to be dependent on the presence of calcium in the medium, however, in the absence of calcium the signal was not completely abolished [in the GL *p*_(0)_ < 0.05 and in the IGL *p*_(0.001)_ < 0.05]. The dependence on calcium is an indication that the signal was predominantly synaptic in nature (as opposed to antidromic, for example). Next we wanted to know which synapses were contributing to this response. The non-NMDA glutamate receptor blocker CNQX (12 μM) was found to also reduce the signal significantly [in the GL *p*_(0.019)_ < 0.05 and in the IGL *p*_(0.02)_ < 0.05], whilst the selective NMDA receptor antagonist D-APV (20 μM) failed to do so [in the GL *p*_(0.926)_ > 0.05 and in the IGL *p*_(0.526)_ > 0.05], indicating that the signal recorded in the GL and the IGL, in response to single pulse stimulation of the VPM, was predominantly the result of non-NMDA glutamatergic synapses. Finally, in view of the extensive inhibitory networks present at the thalamocortical synapse, we wanted to know to what extent these responses would be modulated by the endogenous inhibitory neurotransmitter GABA. In the presence of GABA the responses were again significantly reduced, although not completely abolished [in the GL *p*_(0.001)_ < 0.05 and in the IGL *p*_(0.001)_ < 0.05]. Bicuculline and picrotoxin, GABA_*A*_ receptor antagonists, both resulted in epileptiform events in S1 (as in e.g., Ma et al., 2004 data not shown).

### Short-term plasticity

**Figure 4:**
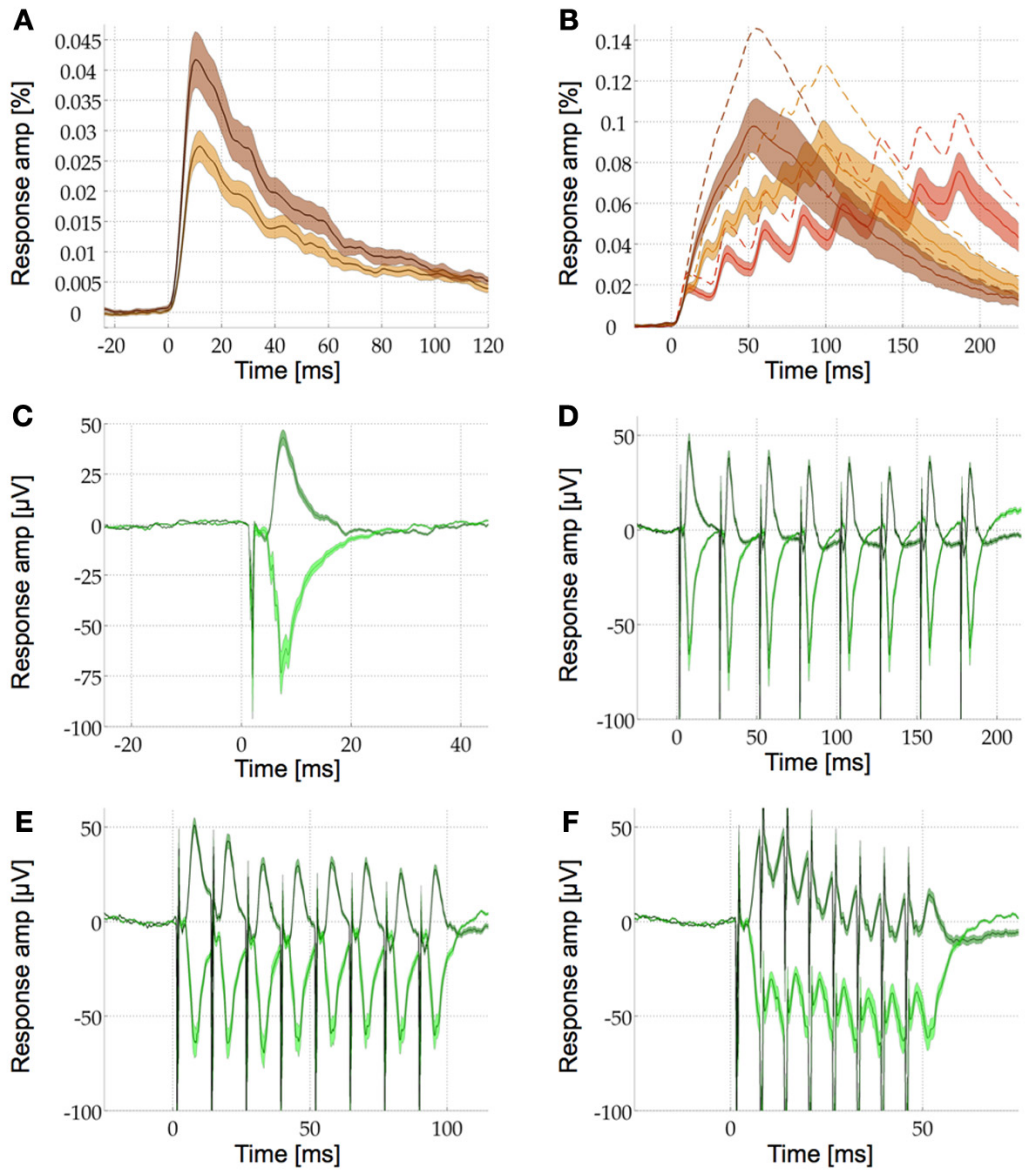
**Comparison of VSDI and LFP response profiles to stimulation with single pulses and pulse trains. (A)** Single pulse response profile in the GL (brown) and the IGL (beige). See Results for details, the shaded area is the s.e.m. **(B)** IGL VSDI response profile for pulse train stimulation with eight pulses at 40 Hz (red), 80 Hz (beige), and 160 Hz (brown). With increasing stimulation frequency the response amplitude increases and the individual pulses sum up into a smooth response envelope at 160 Hz. For visibility only the IGL is shown in detail [same format as in **(A)**], the mean responses in the GL are shown with dotted lines only (the s.e.m. are similar for the two layers).**(C)** Single pulse LFP responses in the GL (dark green) and the IGL [light green, same format as in **(A)**]. The opposite polarity of the response in the two layers can be seen as a positive or a negative deflection after the stimulation artifact (sharp negative discontinuity). **(D–F)** LFP response profile for pulse train stimulation with eight pulses at 40 Hz **(D)**, 80 Hz **(E)**, and 160 Hz **(F)**, same format as in **(C)**. In comparison to **(B)** the responses return to baseline faster so the individual pulses can still be seen at 160 Hz, albeit broken up by the stimulation artifacts.

In a final experiment we investigated the plasticity of the VSDI response and the LFP in S1. We plotted both the response to single pulse stimulation as well as the response to a train of 8 pulses at various frequencies for the VSDI (Figures 4A,B) and the LFP signal (Figures 4C–F). In response to single pulse stimulation both the VSDI and the LFP signal showed a single peak with a fast attack and a slower decay. The VSDI peak was significantly smaller in the IGL than in the GL (Figure 4A, see above and Figure 3A for quantification). The LFP was of opposite polarity in the IGL. It was also non-significantly earlier then in the GL (*t* = 7.2 ms, *p* = 0.523 > 0.05) but of significantly higher absolute amplitude (*m* = −73.59 μV ± 10.49) than in the GL (*t* = 7.6 ms, *m* = 43.13 μV ± 3.8, *p* = 0.0095 < 0.05, Figure 4C). Stimulation with pulse trains of 8 pulses at 40, 80, and 160 Hz resulted in a train of peaks of different amplitudes in both VSDI and LFP signals although the individual peaks could not be distinguished at 160 Hz in the VSDI recordings (Figures 4B,D–F). Due to the shorter time course of the LFP, the signal returned close to baseline in between the individual stimulation pulses at 40 and 80 Hz(Figures 4D,E *cf*. 4F). In the VSDI recordings at all three frequencies the signal was summed up to form a growing response envelope that reached higher than the individual underlying pulses that formed it. The maximal response amplitudes and their time points for these VSD response envelopes were (mean, s.e.m., and *t*) in the GL (0.106% ± 0.122, 187 ms)_40 Hz_; (0.131% ± 0.153, 99 ms)_80 Hz_; (0.148% ± 0.172, 55 ms)_160 Hz_ and in the IGL (0.078% ± 0.087, 186 ms)_40 Hz_; (0.091% ± 0.103, 98 ms)_80 Hz_; (0.1% ± 0.114, 54 ms)_160 Hz_ (Figure 4B, *n* = 20).

**Figure 5:**
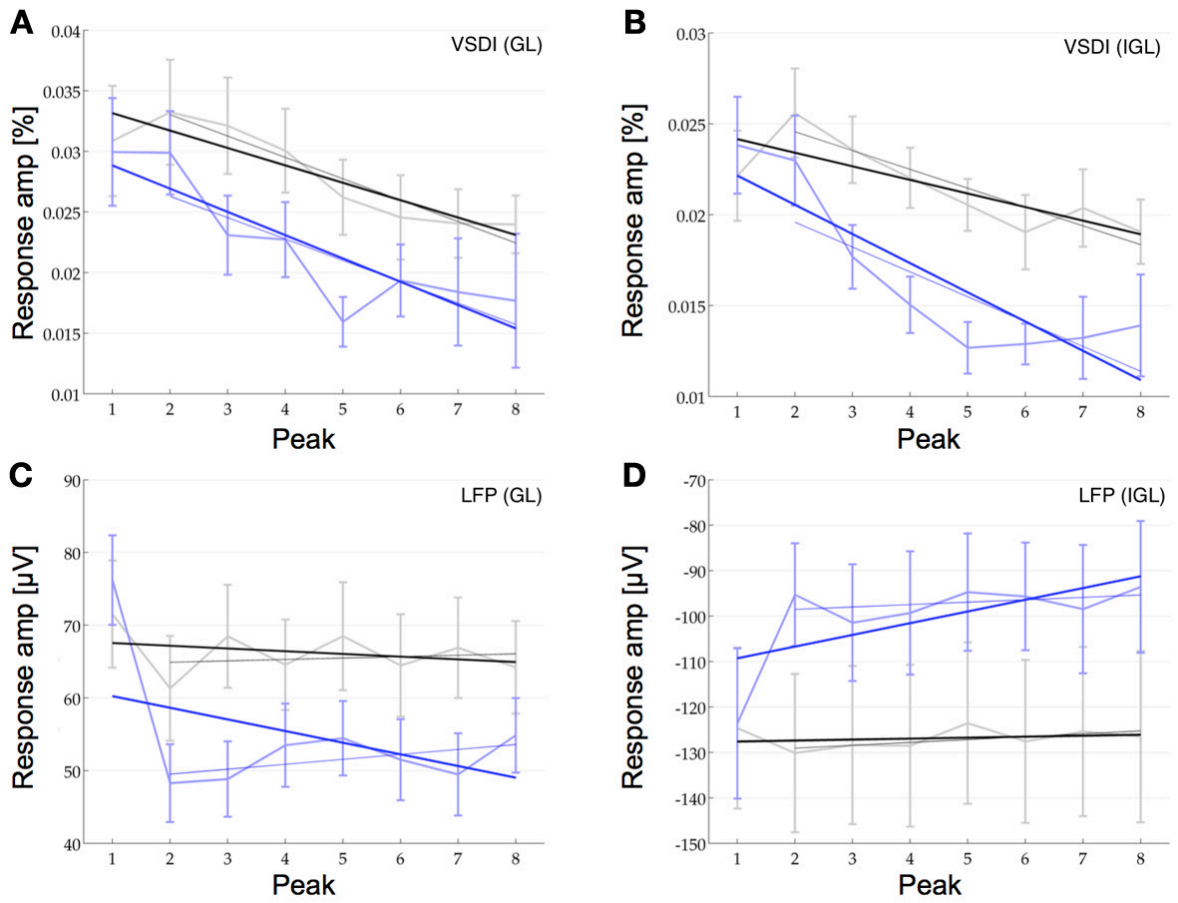
**Short-term plasticity at the population level**. The response amplitude of the individual peaks at 40 Hz (black) and at 80 Hz (blue) compared to their relative baseline are plotted for the VSDI response in the GL **(A)** and the IGL **(B)** and for the LFP in the GL **(C)** and the IGL **(D)** (note the negative y-axis in **D**!). Linear curve fitting over all eight pulses shows depression in all four plots (thick lines with negative slopes, see Results for details). Despite this overall depression, in the VSDI responses **(A,B)**, the second pulse seems to be facilitated over the first at 40 Hz (higher amplitude of second data point over first). Curve fitting over only the last seven pulses (light lines), also revealed, that in the LFP the remaining 7 pulses showed hardly any short-term plasticity at all (**C,D**, *n* = 16).

We then compared how the amplitudes of the individual peaks within these pulse trains change in the VSDI and the LFP signal at 40 and 80 Hz (at 160 Hz the response envelope prohibited this type of analysis). For this we fitted a linear function to the relative amplitudes of the individual peaks (i.e., the maximum of each peak as compared to its local baseline, see Methods for details). These fitted linear functions revealed depression in the LFP and the VSDI signal in both layers. The slopes of this depression in the LFP were −3.8%_40 Hz_ and −18.6%_80 Hz_ in the GL and −1.2%_40 Hz_ and −16.5%_80 Hz_ in the IGL. In the VSDI signal the slopes were −30.3%_40 Hz_ and −46.6%_80 Hz_ in the GL and −21.7%_40 Hz_ and −50.8%_80 Hz_ in the IGL (Figure 5, *n* = 16, note the negative y-axis in Figure 5D). However, when looking at the individual peaks in further detail we saw, what seemed like a more complex pattern of short-term plasticity. Most notably the difference between the first and the second peak, in some of the conditions, seemed different to the differences between all following peaks.

In the VSDI data at 40 Hz the second peak showed some degree of facilitation over the first by 7.7% in the GL and by 15.6% in the IGL. At 80 Hz, on the other hand, it still seemed to be marginally depressed by −0.2% in the GL and by −3.6% in the IGL, but even here, the depression seemed less strong than for the following few peaks. In the LFP data we saw the opposite effect. Here the second peak was highly depressed over the first one at 80 Hz by −36.6% in the GL and by −22.9% in the IGL and also at 40 Hz, at least in the GL by −14.3%, whilst in the IGL we did not observe depression but instead some marginal facilitation by 4.4% (Figure 5). Note, however, the comparatively large error bars, which resulted from the fact that the data is relative (i.e., the square root of the sum of the squared errors of the measured quantity *and* it's relative baseline). Under these conditions only the depression between the first two peaks in the LFP of the GL at 80 Hz could be shown to be significant [Bonferroni corrected *p*_(0.0018)_ < 0.0125]. In view of the fact that the dynamics between the first two peaks seemed, at least to some extent, different, we then fitted a second linear function to only the 2nd through 8th peak. In the VSDI signal this fit to the remaining 7 peaks still showed depression as was originally observed across all peaks (−37.4%_40 Hz_ and −47%_80 Hz_ in the GL and −29.5%_40 Hz_ and −48.9%_80 Hz_ in the IGL). In the LFP signal, however, the depression changed into an almost flat profile with only slight facilitation in the GL 2.1%_40 Hz_ and 9.6%_80 Hz_ and just a slight depression in the IGL with slopes of −3.4%_40 Hz_ and −3.8%_80 Hz_.

The data shows that in response to a train of pulses at 40 Hz the VSDI response in both layers displayed some extent of facilitation between the first and the second peak followed by depression for the following peaks. The LFP signal, on the other hand, showed depression between the first two peaks (with the exception of the IGL at 40 Hz) in both layers followed by almost no plasticity for the following seven peaks (see Discussion).

## Discussion

### Barrel anatomy in the IGL

Upon exiting the VPM thalamocortical axons run caudally within subcortical white matter before rising up inside a barrel along the radial axis and synapsing in the GL with collaterals in the IGL. The DiI stained slices reflected this local anatomy. They also revealed the barrel structure of the GL with increased staining in the barrel lumen compared to the barrel walls. To our surprise, we also saw the same blobs of DiI stain in the IGL. Whilst there is some evidence, that the barrel structure is maintained in layer II/III (Kim and Ebner, 1999; Shepherd et al., 2005), to our knowledge, the current data marks the first time that a barrel structure has also been shown in the anatomy of the IGL.

### Interpreting the VSDI signal

In the GL and the IGL we observed a VSDI response onset after 4 ms. Although the GL is further away from the thalamus than the IGL, the maximal response amplitude was reached first in the GL. The significantly faster rise time in the GL may result from its role in the fast processing of the main primary sensory input into cortex. The response onset may seem substantially earlier then that reported recently *in vivo* (Constantinople and Bruno, 2013), note however that the reference point here is stimulation in the thalamus, whilst the mentioned *in vivo* study's reference point was the deflection of the whiskers. Indeed our findings correspond to those reported by Constantinople and Bruno, in that we too find earlier activation (time to peak) in the GL but at the same time we also recorded similarly early responses in the IGL in the VSDI signal.

The signal then decayed slowly in both layers over the course of the next 100 ms. Whilst at this point the signal was within the predefined numbers of standard deviations of the pre-stimulus baseline, it was still somewhat elevated (Figure 4A). This low amplitude slow component of the signal is most likely the result of underlying glial activity, which has been observed with VSDI in various preparations alongside the purely neuronal response (Grinvald et al., 1982; Salzberg et al., 1983; Konnerth and Orkand, 1986; Lev-Ram and Grinvald, 1986).

The fact that the VSDI signal was significantly reduced in the absence of calcium implies that its main component was not the result of antidromic activation of cell bodies and dendrites. The fast time to peak in both layers and the fact that at both 40 and 80 Hz the response followed the stimulation pattern closely, further suggests that a significant early part of the recorded signal was probably of monosynaptic origin. Whilst the overall increase of the response envelope measured at 40, 80, and 160 Hz could hence be interpreted as a summation of monosynaptic short-term synaptic plasticity (Zucker and Regehr, 2002), it seems more likely that the increased activation of the cortex under the pulse train paradigm is, at least in part, also the result of a cumulative activation of voltage dependent NMDA receptors (Collingridge and Bliss, 1995; Laaris et al., 2000).

In VSDI each pixel records the average response over a small volume containing axons, dendrites, neuronal cell bodies, and glia. Because the surface area of dendrites will usually be much larger than that of somata or axons, and because the time scale of a response in the dendrites is substantially slower than in axons, we can assume, however, that the recorded signal is primarily of dendritic origin (Yuste et al., 1997). Therefore, we can conclude that our recordings reflect the activity in the postsynaptic arborization of the thalamocortical afferents and that the recorded activity is, to a large extent, made up of an early monosynaptic response as well as a short-term, GABA-dependent local polysynaptic process.

The cell bodies of some of the imaged dendrites may lie far from the actual recording site, hence, the activity in a recording site may not necessarily be directly related to the local cell population as defined by histological staining of cell nuclei. In the GL dendritic arborization is primarily local (Simons and Woolsey, 1984), hence the dimensions of the recorded VSDI signal overlapped with the cytoarchitecture of layer IV. In the IGL cells of both layer V and VI have more extensive and divers dendritic arborizations (Zhang and Deschênes, 1997; Schubert et al., 2001). Here the recorded VSDI activity did not directly correspond to any cytoarchitectonic layer but rather constituted a supervening *functional* cortical layer at the border of layer V and VI. The traditional six layers of cortex are defined on the basis of the distribution of different cell bodies. From a functional point of view, however, the distribution of the computational elements in cortex may be more relevant. We believe the two dendritic networks, the GL and the IGL, described here, may constitute two initial “data processors” in cortex that may stand at the beginning of two parallel computational pathways. To find out more about their characteristics, we next investigated their short-term dynamics.

### Short-term plasticity

Recent work by Viaene et al. (2011a,b) described different classes of short-term plasticity in postsynaptic thalamocortical neurons of the GL and the IGL. Besides purely depressive responses to thalamic pulse train stimulation (termed Class 1A and 1B), they also described a mixed response showing facilitation between the first two excitatory postsynaptic potentials followed by depression for the following peaks (termed Class 1C). At the level of the population response recorded here, thousands of responses from all three classes are orchestrated together. We therefore investigated what the resulting dynamics of the population as a whole might be, as compared to its underlying elements.

When looking at all eight pulses at 40 Hz the VSDI response showed a strong slope of depression whilst the LFP showed slightly weaker depression. At 80 Hz we found depression in both the LFP and the VSDI response in both layers. When looking at the data a little closer we found that, at 40 Hz, there may be facilitation in the VSDI response in both the GL and the IGL between the first two pulses but we could not show this effect to be significant. In the LFP data, however, we found strong depression between the first two peaks at 80 Hz in both layers. The slope across the peaks following this strong inhibitory response were relatively flat across all conditions in the LFP.

The population response is unlikely to show the exact same characteristics as its underlying elements. However, in view of the fact, that a relatively large number of cells in the IGL have been shown to have class 1C responses, it is interesting to see, that also in the population response we saw a difference between the first two pulses and the following six pulses in some of our experiments. In the VSDI response the first two peaks may be facilitated, as in the class 1C response. In the LFP, however, we observed the opposite effect in the form of a strong depression. This difference between the two responses may arise from the different origin of the LFP signal *vs*. the VSDI signal. Whilst the origin of the LFP is primarily a weighted average of synchronized dendro-somatic components of the synaptic signal (Logothetis, 2008), the precise nature of the VSDI signal is still not fully understood. It could also be, for example, that type 1C neurons are differentiated anatomically, as well as synaptically, from other neurons in cortex. For example, if type 1C neurons generally also exhibited more extensive dendritic arborization, it would not be surprising if the VSDI response would reflect the activity of such cells more closely. The same argument would, however, not necessarily be true for the LFP signal. Further research will be needed to investigate the exact nature of these dynamics and how they emerge from the underlying activity at the level of single neurons.

The experiments presented here describe two large networks of dendritic arborization that represent the first level of processing of thalamic input into S1. DiI staining revealed that in both networks the thalamocortical afferents target these networks within the barrel boundaries known from layer IV. The short-term dynamics within these two networks show general depression in response to pulse train stimulation. Within this depression, the relationship of the first two peaks is different under some conditions, as compared to the dynamics between the remaining six peaks.

Together, these findings describe postsynaptic population parameters relevant to the study of early cortical network processing. Through the detailed investigation of the dimensions and characteristics of these dendritic networks we hope to be able to provide tools for further research into these highly relevant and complex large scale neuronal systems.

### Conflict of interest statement

The authors declare that the research was conducted in the absence of any commercial or financial relationships that could be construed as a potential conflict of interest.

## References

[B1] Agmon A. Connors B. (1991). Thalamocortical responses of mouse somatosensory (barrel) cortex *in vitro*. Neuroscience 41, 365–379 10.1016/0306-4522(91)90333-J 1870696

[B2] Beierlein M. Gibson J. R. Connors B. W. (2003). Two dynamically distinct inhibitory networks in layer 4 of the neocortex. J. Neurophysiol. 90, 2987–3000 10.1152/jn.00283.2003 12815025

[B3] Benshalom G. White E. L. (1986). Quantification of thalamocortical synapses with spiny stellate neurons in layer IV of mouse somatosensory cortex. J. Comp. Neurol. 253, 303–314 10.1002/cne.902530303 3793995

[B3a] Constantinople C. M. Bruno R. M. (2013). Deep cortical layers are activated directly by thalamus. Science 340, 1591–1594 10.1126/science.1236425 23812718 PMC4203320

[B4] Collingridge G. L. Bliss T. V. (1995). Memories of NMDA receptors and LTP. Trends Neurosci. 18, 54–56 10.1016/0166-2236(95)80016-U 7537406

[B5] Franks N. P. (2008). General anaesthesia: from molecular targets to neuronal pathways of sleep and arousal. Nat. Rev. Neurosci. 9, 370–386 10.1038/nrn2372 18425091

[B6] Geisler S. Heilmann H. Veh R. W. (2002). An optimized method for simultaneous demonstration of neurons and myelinated fiber tracts for delineation of individual trunco- and palliothalamic nuclei in the mammalian brain. Histochem. Cell Biol. 117, 69–79 10.1007/s00418-001-0357-z 11819099

[B7] Gibson J. R. Beierlein M. Connors B. W. (1999). Two networks of electrically coupled inhibitory neurons in neocortex. Nature 402, 75–79 10.1038/47035 10573419

[B8] Grinvald A. Manker A. Segal M. (1982). Visualization of the spread of electrical activity in rat hippocampal slices by voltage-sensitive optical probes. J. Physiol. 333, 269–291 7182467 10.1113/jphysiol.1982.sp014453 PMC1197248

[B9] Hájos N. Mody I. (2009). Establishing a physiological environment for visualized *in vitro* brain slice recordings by increasing oxygen supply and modifying aCSF content. J. Neurosci. Methods 183, 107–113 10.1016/j.jneumeth.2009.06.005 19524611 PMC2753642

[B10] Higashi S. Crair M. C. Kurotani T. Inokawa H. Toyama K. (1999). Altered spatial patterns of functional thalamocortical connections in the barrel cortex after neonatal infraorbital nerve cut revealed by optical recording. Neuroscience 91, 439–452 10.1016/S0306-4522(98)00666-6 10366001

[B11] Hill M. R. H. Greenfield S. A. (2011). The membrane chamber: a new type of *in vitro* recording chamber. J. Neurosci. Methods 195, 15–23 10.1016/j.jneumeth.2010.10.024 21075142

[B12] Itami C. Samejima K. Nakamura S. (2001). Improved data processing for optical imaging of developing neuronal connectivity in the neonatal mouse barrel cortex. Brain Res. Brain Res. Protoc. 7, 103–114 10.1016/S1385-299X(01)00048-4 11356376

[B13] Kim U. Ebner F. F. (1999). Barrels and septa: separate circuits in rat barrels field cortex. J. Comp. Neurol. 408, 489–505 10.1002/(SICI)1096-9861(19990614)408:4<489::AID-CNE4>3.0.CO;2-E 10340500

[B14] Konnerth A. Orkand R. K. (1986). Voltage-sensitive dyes measure potential changes in axons and glia of the frog optic nerve. Neurosci. Lett. 66, 49–54 10.1016/0304-3940(86)90164-3 3487054

[B14a] Laaris N. Carlson G. Keller A. (2000). Thalamic-evoked synaptic interactions in barrel cortex revealed by optical imaging. J. Neurosci. 20, 1529–1537 10662842 10.1523/JNEUROSCI.20-04-01529.2000 PMC6772365

[B15] Laaris N. Keller A. (2002). Functional independence of layer IV barrels. J. Neurophysiol. 87, 1028–1034 10.1152/jn.00512.2001 11826066 PMC2803341

[B16] Land P. W. Simons D. J. (1985). Cytochrome oxidase staining in the rat SmI barrel cortex. J. Comp. Neurol. 238, 225–235 10.1002/cne.902380209 2413086

[B17] Lev-Ram V. Grinvald A. (1986). Ca2+- and K+-dependent communication between central nervous system myelinated axons and oligodendrocytes revealed by voltage-sensitive dyes. Proc. Natl. Acad. Sci. U.S.A. 83, 6651–6655 10.1073/pnas.83.17.6651 2428038 PMC386562

[B18] Llinás R. R. Leznik E. Urbano F. J. (2002). Temporal binding via cortical coincidence detection of specific and nonspecific thalamocortical inputs: a voltage-dependent dye-imaging study in mouse brain slices. Proc. Natl. Acad. Sci. U.S.A. 99, 449–454 10.1073/pnas.012604899 11773628 PMC117580

[B19] Logothetis N. K. (2008). What we can do and what we cannot do with fMRI - Suplementary material. Nature 453, 869–878 10.1038/nature06976 18548064

[B20] Ma H. Wu C. Wu J. (2004). Initiation of spontaneous epileptiform events in the rat neocortex *in vivo*. J. Neurophysiol. 91, 934–945 10.1152/jn.00274.2003 14534285 PMC2909741

[B21] Pierret T. Lavallée P. Deschênes M. (2000). Parallel streams for the relay of vibrissal information through thalamic barreloids. J. Neurosci. 20, 7455–7462 Available online at: http://www.jneurosci.org/content/20/19/7455.short 11007905 10.1523/JNEUROSCI.20-19-07455.2000 PMC6772772

[B22] Porter J. T. Johnson C. K. Agmon A. (2001). Diverse types of interneurons generate thalamus-evoked feedforward inhibition in the mouse barrel cortex. J. Neurosci. 21, 2699–2710 Available online at: http://www.jneurosci.org/content/21/8/2699.short 11306623 10.1523/JNEUROSCI.21-08-02699.2001 PMC6762510

[B23] Ren J. Q. Aika Y. Heizmann C. W. Kosaka T. (1992). Quantitative analysis of neurons and glial cells in the rat somatosensory cortex, with special reference to GABAergic neurons and parvalbumin-containing neurons. Exp. Brain Res. 92, 1–14 10.1007/BF00230378 1486945

[B24] Salzberg B. M. Obaid A. L. Senseman D. M. Gainer H. (1983). Optical recording of action potentials from vertebrate nerve terminals using potentiometric probes provides evidence for sodium and calcium components. Nature 306, 36–40 10.1038/306036a0 6633657

[B25] Schubert D. Staiger J. F. Cho N. Kotter R. Zilles K. Luhmann H. J. (2001). Layer-specific intracolumnar and transcolumnar functional connectivity of layer V pyramidal cells in rat barrel cortex. J. Neurosci. 21, 3580–3592 Available online at: http://www.jneurosci.org/content/21/10/3580.short 11331387 10.1523/JNEUROSCI.21-10-03580.2001 PMC6762473

[B26] Shepherd G. M. G. Stepanyants A. Bureau I. Chklovskii D. Svoboda K. (2005). Geometric and functional organization of cortical circuits. Nat. Neurosci. 8, 782–790 10.1038/nn1447 15880111

[B27] Simons D. J. Woolsey T. A. (1984). Morphology of Golgi-Cox-impregnated barrel neurons in rat SmI cortex. J. Comp. Neurol. 230, 119–132 10.1002/cne.902300111 6512012

[B28] Tan Z. Hu H. Huang Z. J. Agmon A. (2008). Robust but delayed thalamocortical activation of dendritic-targeting inhibitory interneurons. Proc. Natl. Acad. Sci. U.S.A. 105, 2187–2192 10.1073/pnas.0710628105 18245383 PMC2538896

[B29] Urbano F. J. Leznik E. Llinás R. R. (2007). Modafinil enhances thalamocortical activity by increasing neuronal electrotonic coupling. Proc. Natl. Acad. Sci. U.S.A. 104, 12554–12559 10.1073/pnas.0705087104 17640897 PMC1925036

[B30] Viaene A. N. Petrof I. Sherman S. M. (2011a). Synaptic properties of thalamic input to layers 2/3 and 4 of primary somatosensory and auditory cortices. J. Neurophysiol. 105, 279–292 10.1152/jn.00747.2010 21047937 PMC3023380

[B31] Viaene A. N. Petrof I. Sherman S. M. (2011b). Synaptic properties of thalamic input to the subgranular layers of primary somatosensory and auditory cortices in the mouse. J. Neurosci. 31, 12738–12747 10.1523/JNEUROSCI.1565-11.2011 21900553 PMC3178048

[B32] Wong-Riley M. (1979). Changes in the visual system of monocularly sutured or enucleated cats demonstrable with cytochrome oxidase histochemistry. Brain Res. 171, 11–28 10.1016/0006-8993(79)90728-5 223730

[B33] Woolsey T. A. Van der Loos H. (1970). The structural organization of layer IV in the somatosensory region (SI) of mouse cerebral cortex. The description of a cortical field composed of discrete cytoarchitectonic units. Brain Res. 17, 205–242 10.1016/0006-8993(70)90079-X 4904874

[B34] Yuste R. Tank D. Kleinfeld D. (1997). Functional study of the rat cortical microcircuitry with voltage-sensitive dye imaging of neocortical slices. Cereb. Cortex 7, 546–558 10.1093/cercor/7.6.546 9276179

[B35] Zhang Z. W. Deschênes M. (1997). Intracortical axonal projections of lamina VI cells of the primary somatosensory cortex in the rat: a single-cell labeling study. J. Neurosci. 17, 6365–6379 9236245 10.1523/JNEUROSCI.17-16-06365.1997 PMC6568349

[B36] Zucker R. S. Regehr W. G. (2002). Short-term synaptic plasticity. Annu. Rev. Physiol. 64, 355–405 10.1146/annurev.physiol.64.092501.114547 11826273

